# Statin Therapy Protects White Matter in Cerebral Small Vessel Disease Independent of Lipid‐Lowering: Biomarker and Neuroimaging Evidence

**DOI:** 10.1002/brb3.71359

**Published:** 2026-05-11

**Authors:** Rui Wang, Juluo Chen, Peiqi Ma, Lu Liu, Youmeng Wang, Qiqiang Tang

**Affiliations:** ^1^ The First Affiliated Hospital of USTC, Division of Life Sciences and Medicine University of Science and Technology of China Hefei Anhui China; ^2^ Department of Neurology Fuyang People's Hospital Fuyang Anhui China

## Abstract

**Background:**

Cerebral small vessel disease (CSVD) is characterized by white matter damage, but the roles of hyperlipidemia and statin therapy in its pathogenesis remain controversial. This study aimed to investigate the independent effects of statin therapy and hyperlipidemia on white matter integrity in CSVD patients.

**Objective:**

To investigate the independent effects of hyperlipidemia and statin therapy on white matter damage in CSVD by examining their relationship with serum myelin basic protein (MBP) levels, Fazekas scores, and hippocampal structure.

**Methods:**

This study recruited 141 participants from Fuyang City, China, with 71 CSVD patients and 70 NCSVD controls. Participants underwent multimodal cranial MRI, cognitive assessments, and blood tests. Statistical analyses explored the relationships between MBP levels, Fazekas scores, hippocampal fimbria volume, and statin therapy.

**Results:**

Statin therapy was significantly associated with lower Fazekas scores (p = 0.018), decreased MBP levels (p = 0.006), and higher hippocampal fimbria volumes (p = 0.009 for left fimbria, p = 0.010 for right fimbria) only in the CSVD group. Hyperlipidemia and blood lipid levels showed no statistically significant associations with Fazekas scores, MBP levels, or hippocampal fimbria volumes in either group.

**Conclusion:**

This study demonstrates that statin therapy is independently associated with reduced white matter damage in CSVD, irrespective of peripheral lipid levels. This protective effect is specific to CSVD patients, possibly related to statin therapy improving dysregulated central nervous system lipid metabolism following blood‐brain barrier dysfunction. These findings provide new insights into CSVD pathophysiology and highlight the potential of statins as a therapeutic strategy for white matter protection in CSVD.

## Graphical abstract

This study, through an analysis of neuroimaging markers, biomarkers of white matter damage, and the volume of the hippocampal fimbria (a white matter structure) in patients with cerebral small vessel disease (CSVD), revealed that the protective effect of statin therapy against white matter damage in CSVD patients is independent of its lipid‐lowering effects.

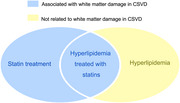

## 1 Introduction

Cerebral small vessel disease (CSVD) represents a complex group of pathological processes primarily affecting the small arteries, arterioles, capillaries, and small veins in the brain. The disease was previously characterized by neuroimaging features including recent small subcortical infarcts, lacunes, white matter hyperintensities (WMH), enlarged perivascular spaces, cerebral microbleeds, and brain atrophy (Wardlaw et al. 2013), as well as cortical superficial siderosis and cortical cerebral microinfarcts (Duering et al. 2023). White matter damage, a cardinal manifestation of CSVD, typically presents as WMH on neuroimaging and is characterized by progressive axonal atrophy and myelin loss (Pantoni 2002). These structural changes frequently correlate with alterations in myelin basic protein (MBP) levels, reflecting the dynamic nature of white matter integrity (Bjerke et al. 2014). Synthesized by oligodendrocytes, MBP plays a crucial role in myelin surface adhesion. Importantly, changes in MBP levels have emerged as a reliable marker for white matter damage in CSVD patients, providing valuable insights into the disease progression (Min et al. 2009).

Among the established risk factors for CSVD, advanced age and hypertension demonstrate the strongest associations (Hilal et al. 2017). While other vascular risk factors such as diabetes, smoking, and hypercholesterolemia have been implicated in CSVD development, their precise roles, particularly that of hypercholesterolemia, remain subjects of intensive debate (Wang et al. 2021). The relationship between hypercholesterolemia and CSVD presents a particularly intriguing paradox in current literature. Several experimental and clinical studies have demonstrated that hypercholesterolemia can induce CSVD development through various mechanisms, including endothelial dysfunction, inflammation, and oxidative stress (Kraft et al. 2017). This is further supported by evidence showing increased CSVD prevalence among patients with familial hypercholesterolemia, suggesting a potential causal relationship (Todate et al., 2019). However, contradictory findings have emerged, with some studies reporting that hypercholesterolemia might be associated with reduced CSVD risk (Ohwaki et al. 2013). Some researchers have even proposed a potential protective effect of moderate hyperlipidemia on cerebral small vessel integrity (Warsch and Wright 2010). This contradiction highlights the complexity of lipid metabolism in cerebral small vessel pathophysiology and suggests the involvement of additional regulatory factors.

Statin therapy, while primarily used for hyperlipidemia treatment, has emerged as a critical factor in CSVD research, demonstrating relatively consistent beneficial effects independent of its lipid‐lowering action. Multiple longitudinal studies indicate that statin therapy can effectively decelerate the progression of primary CSVD imaging features, including WMH, lacunes, and enlarged perivascular spaces (Guo et al. 2020; Ji et al. 2018). Despite concerns regarding potential side effects such as microbleeding (Wu and Chen 2016), the overall evidence strongly supports a protective role of statins in CSVD pathogenesis. This therapeutic effect of statins may significantly confound the assessment of hypercholesterolemia's impact on CSVD, necessitating independent analysis of these factors to clarify their respective roles.

The heterogeneous nature of CSVD presents unique challenges in studying its risk factors and progression. Even hypertension, the most well‐established risk factor, shows varying associations with disease progression across different age groups (van Dijk et al. 2008). Furthermore, the complex interplay between vascular risk factors, blood‐brain barrier integrity, and white matter pathology remains poorly understood. To address these challenges, we designed a study focusing on community‐dwelling individuals with preserved cognitive function and without overt neurological signs, identifying CSVD through comprehensive imaging examinations. Given our study population's carefully restricted nature based on symptoms and cognitive status, resulting in relatively mild brain structural lesions, we selected MBP levels as a quantitative marker for white matter damage. This strategy enables us to detect subtle changes in white matter integrity before the development of significant structural abnormalities. Through this comprehensive approach, we aim to clarify the complex relationship between statin therapy, hyperlipidemia, and white matter damage in CSVD, while exploring the potential mechanisms involving blood‐brain barrier dysfunction and cortical structural changes. These findings may provide crucial insights for optimizing therapeutic strategies in CSVD management and prevention.

## 2 Materials and Methods

### 2.1 Study Design and Population

This cross‐sectional study was conducted at the Fuyang People's Hospital, Anhui Province, China, from May to June 2023. The study protocol was approved by the Ethics Committee of Fuyang People's Hospital (Medical [2022] No. 26) and adhered to the principles of the Declaration of Helsinki. All participants provided written informed consent prior to enrollment.

**FIGURE 1:**
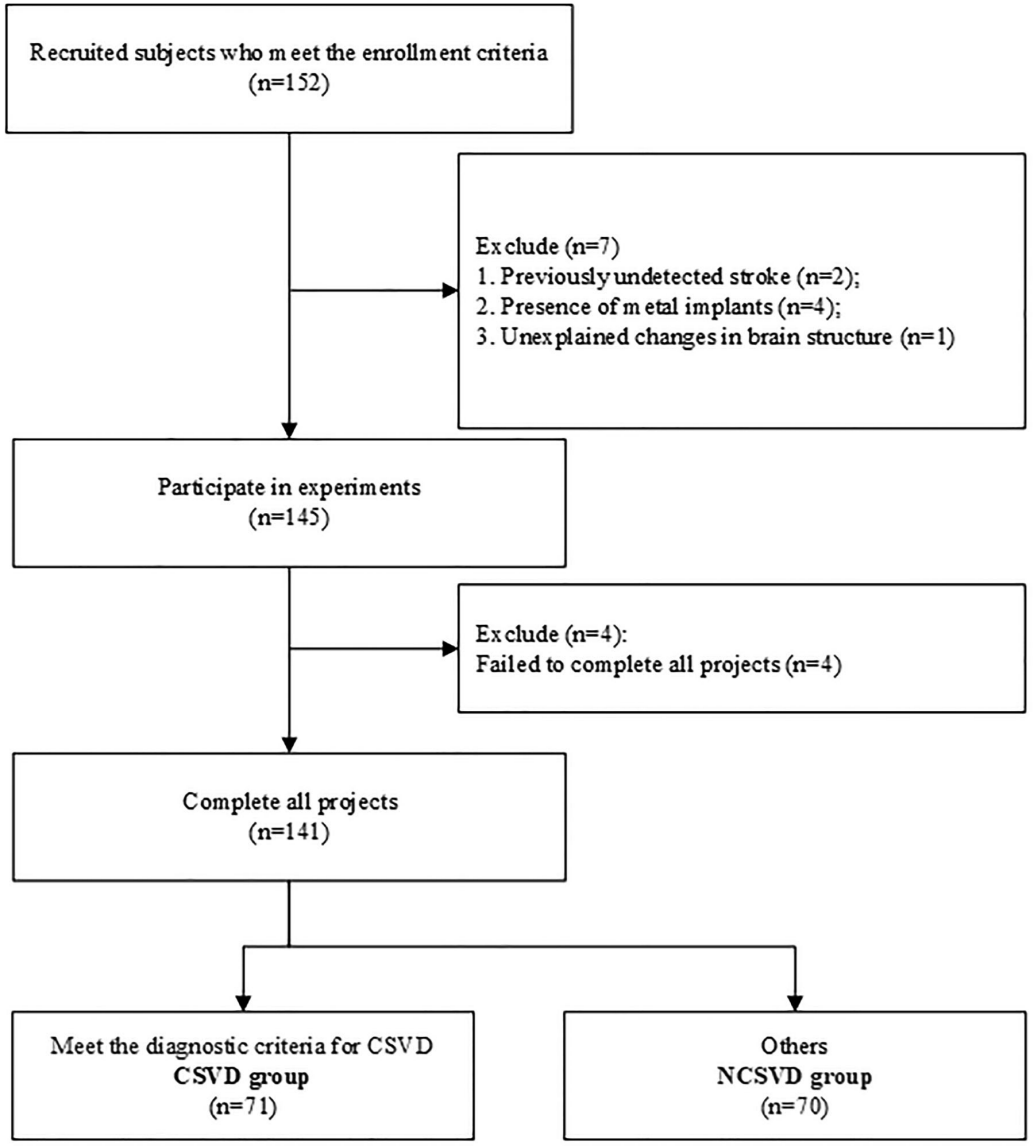
Recruitment process for subjects.

A total of 152 community‐dwelling individuals from Fuyang City, Anhui Province, China, were initially screened for eligibility. After applying the exclusion criteria, 141 subjects were included in the final analysis. Based on neuroimaging criteria, participants were classified into two groups: 71 individuals in the CSVD group and 70 in the NCSVD group. (Figure 1)

### 2.2 Participant Selection

Community‐dwelling participants were recruited through local health centers using the following inclusion criteria: (1) age between 50 and 75 years; (2) no history of clinical stroke; (3) absence of obvious CSVD‐related symptoms (cognitive impairment, gait disturbance, psychiatric symptoms, or sleep disorders); (4) normal liver and kidney function (serum creatinine < 1.5 mg/dL, ALT and AST < 2.5 times upper limit of normal); (5) no contraindications to MRI; (6) no long‐term use of antibiotics or antiplatelet agents within the past three months; and (7) ability to complete multiple MRI sessions.

Exclusion criteria comprised: (1) history of brain tumor, trauma, central nervous system infection, thyroid dysfunction, vitamin B1/B12 deficiency, alcoholic brain injury, syphilis, or malnutrition; (2) severe psychiatric illness or symptoms affecting cognitive assessment; (3) large vessel infarction, severe hemorrhagic stroke, or WMH due to other causes (leukodystrophy, inflammatory demyelinating diseases, or vasculitis); (4) active severe infection; (5) severe microbleed burden on susceptibility‐weighted imaging (SWI); or (6) any condition deemed unsuitable for study participation by the investigators.

Participants were classified into CSVD and NCSVD groups based on standardized neuroimaging criteria according to STRIVE v1 (Wardlaw et al. 2013). CSVD diagnosis required either (1) moderate‐to‐severe white matter lesions (Fazekas score > 1 in deep or > 2 in periventricular regions) or (2) mild white matter hyperintensity (Fazekas score = 1 in deep or ≤ 2 in periventricular regions) combined with ≥ 1 lacunar infarct or > 3 microbleeds. The final diagnosis was confirmed through consensus review by a multidisciplinary panel including two neuroradiologists and two cerebrovascular specialists, with discrepancies resolved through consultation with a senior imaging expert.

Hypertension was defined as subjects with a systolic blood pressure greater than 140 mmHg or a diastolic blood pressure greater than 90 mmHg at rest and subjects who were currently taking antihypertensive drugs. Diabetes was defined as subjects with a fasting blood glucose greater than 7 mmol/L and a glycated hemoglobin greater than 6.5% and subjects who were currently taking antihyperglycemic drugs. Smoking history was defined as still smoking or smoking in the past but quitting within 3 years. Drinking history was defined as ethanol consumption ≥ 40 g/d for men and ≥ 20 g/d for women. Hyperlipidemia was defined as total cholesterol ≥ 6.2 mg/L, low‐density lipoprotein cholesterol ≥ 4.1 mg/L, or triglycerides ≥ 2.3 mg/L according to the 2023 China Guidelines for Lipid Management (Li et al. 2023). Statin therapy was defined as continuous statin therapy for more than 3 months.

### 2.3 MRI Protocol and Analysis

All participants underwent multimodal MRI using a 3.0‐T Philips Ingenia CX system (Philips Healthcare, Best, Netherlands) with a 32‐channel head coil. The imaging protocol included: Structural sequences: 3D T1‐weighted imaging: TR/TE = 7.9/3.5 ms, slice thickness = 1 mm, no gap, 140 slices; T2‐weighted imaging: TR/TE = 3000/90 ms, slice thickness = 5 mm, no gap, 24 slices; SWI: TR/TE = 31/7.2 ms, slice thickness = 5.6 mm, no gap, 48 slices.

WMH was quantified using the Fazekas scale (Fazekas et al. 1987); lacunes were evaluated by direct counting; enlarged perivascular spaces (ePVS) were graded separately in the basal ganglia (BG) and centrum semiovale (CSO) (Potter et al. 2015) using a visual semiquantitative ordinal scale; and cerebral microbleeds (CMBs) were scored according to the MARS scale (Gregoire et al. 2009). Two experienced neuroradiologists (with 10 and 15 years of experience, respectively) performed the ratings independently, blinded to clinical information. Discrepancies were resolved through consensus discussion with a third senior neuroradiologist (with 20 years of experience). Structural brain analysis, including hippocampal subfield volumetry, was performed using FreeSurfer software (version 7.4.1) following standard procedures.

### 2.4 Neuropsychological Assessments

Cognitive function was evaluated using the Chinese version of the Montreal Cognitive Assessment Basic (MoCA‐BC) scale. Three trained investigators conducted all assessments after completing standardized training and inter‐rater reliability testing (κ > 0.85). The MoCA‐BC was specifically selected for its validation in elderly Chinese individuals with lower education levels. Dementia was excluded using education‐adjusted cutoff values: ≤ 19 for illiterate, ≤ 22 for primary school, and ≤ 24 for junior high school and above (Chen et al. 2016). All subjects included in the statistical analysis were scored within the normal range.

### 2.5 Laboratory Analysis

Fasting blood samples (3 mL) were collected between 7:00‐9:00 a.m., allowed to clot for 30 min at room temperature, and centrifuged at 3000 g for 10 min. Serum was aliquoted and stored at −80°C until analysis. MBP levels were measured using commercially available ELISA kits (Abcam, Cambridge, UK) according to the manufacturer's instructions. The inter‐ and intra‐assay coefficients of variation were < 10%. Routine biochemical parameters were analyzed using standard automated techniques in the hospital's clinical laboratory.

### 2.6 MRI Data Processing

Hippocampal subfield volumetry was performed using FreeSurfer software (version 7.4.1) following the standard recon‐all pipeline with the additional hippocampal subfield segmentation. The processing steps included: Motion correction and conforming to 1 mm isotropic space; Non‐uniform intensity normalization, Talairach transformation, skull stripping, segmentation of subcortical white matter, and deep gray matter structures, hippocampal subfield segmentation using a statistical atlas built primarily upon ultra‐high resolution (∼0.1 mm isotropic) ex vivo MRI data.

Quality control was performed at each step by visual inspection. Cases with significant artifacts or processing failures were reprocessed or excluded if corrections were unsuccessful.

### 2.7 Statistical Analysis

Statistical analyses were performed using IBM SPSS Statistics version 24.0 (IBM Corp., Armonk, NY). Data normality was assessed using the Kolmogorov–Smirnov test. Normally distributed continuous variables are presented as mean ± standard deviation and compared using the *t*‐test to account for unequal group sizes. Non‐normally distributed variables are presented as median (interquartile range) and compared using the Mann–Whitney *U* test. Categorical variables are presented as frequencies (percentages) and compared using the chi‐square test when appropriate.

Binary logistic regression was used for categorical variables and linear regression for continuous variables. Two‐sided *p*‐values < 0.05 were considered statistically significant.

## 3 Results

### 3.1 Demographic and Clinical Characteristics

A total of 141 participants were included in the study, divided into two groups: CSVD (*n* = 71) and NCSVD (*n* = 70). The CSVD group showed significantly higher age (median 65 vs. 57 years, p < 0.001) and lower education levels (median 9 vs. 12 years, p < 0.001) compared to the NCSVD group. The CSVD group had a higher prevalence of hypertension (56.3% vs. 32.9%, p = 0.005) and was more likely to be on statin therapy (33.8% vs. 8.6%, p < 0.001). Initially, there was a significant difference in MoCA‐BC scores between the groups (median 25 vs. 26, p < 0.001). However, after adjusting for age, this difference in MoCA‐BC scores lost statistical significance (F = 1.455, p = 0.230).

While not statistically significant, the CSVD group showed trends toward higher rates of diabetes (16.9% vs. 10.0%, p = 0.230), smoking (35.2% vs. 21.4%, p = 0.069), and alcohol consumption (43.7% vs. 34.3%, p = 0.254). There were no significant differences between groups in gender distribution, prevalence of hyperlipidemia, or in blood pressure and lipid profile measurements (MBP, TC, TG, HDL‐C, and LDL‐C) (Table 1).

**TABLE 1:** Demographic and clinical characteristics.

Characteristics	CSVD (*n* = 71)	NCSVD (*n* = 70)	*χ* ^2^/t/Z	p
Age (years)	65(12)	57(6)	−4.296	< 0.001
Education (years)	9(6)	12(3.75)	−3.778	< 0.001
Male, *n* (%)	32 (45.1%)	28 (40.0%)	0.371	0.543
Hypertension, *n* (%)	40 (56.3%)	23 (32.9%)	7.863	0.005
Diabetes, *n* (%)	12 (16.9%)	7 (10.0%)	1.440	0.230
Smoking, *n* (%)	25 (35.2%)	15 (21.4%)	3.295	0.069
Drinking, *n* (%)	31 (43.7%)	24 (34.3%)	1.302	0.254
Statin therapy, n (%)	24 (33.8%)	6 (8.6%)	13.397	< 0.001
Hyperlipidemia, n (%)	20 (28.2%)	19 (27.1%)	0.019	0.892
MBP (ng/mL)	6.57 ± 1.66	6.41 ± 1.87	0.512	0.609
TC (mmol/L)	5.00 ± 1.06	5.26 ± 0.88	−1.604	0.111
TG (mmol/L)	1.26(1.02)	1.20(1.07)	−0.596	0.551
HDL‐C (mmol/L)	1.30(0.45)	1.34(0.36)	−0.878	0.380
LDL‐C (mmol/L)	2.62±0.78	2.87±0.93	−1.763	0.080
MoCA‐BC	25(4)	26(3)	−3.515	< 0.001

Abbreviations: LDL‐C, Low density lipoprotein cholesterol; MoCA‐BC, Montreal cognitive assessment basic edition score; MBP, Myelin basic protein; TC, Total cholesterol; TG, Triglycerides;.

### 3.2 Effects of Statin Therapy and Hyperlipidemia on CSVD Patients

Based on whether patients were using statins and whether hyperlipidemia was present, we further categorized the CSVD group. We found that in the statin therapy subgroup, there were significant differences in MBP levels (*p* = 0.003) and the prevalence of hypertension (*p* = 0.001). In contrast, when grouping based on the presence of hyperlipidemia, apart from lipid profile parameters, no significant differences were observed in any other characteristics between subgroups (Table 2).

**TABLE 2:** Comparison of clinical characteristics and biomarkers in CSVD patients stratified by statin therapy and hyperlipidemia status.

	Statin therapy		Hyperlipidemia	
Characteristics	*χ* ^2^/t/Z	p	*χ* ^2^/t/Z	p
Age (years)	−0.786	0.432	−0.045	0.964
Education (years)	−0.939	0.348	−0.197	0.843
Male, *n* (%)	0.839	0.360	0.289	0.591
Hypertension, *n* (%)	10.741	0.001	1.455	0.228
Diabetes, *n* (%)	1.693	0.193	0.944	0.331
Smoking, *n* (%)	0.662	0.416	1.273	0.259
Drinking, *n* (%)	0.059	0.809	0.849	0.357
MBP (ng/mL)	−3.051	0.003	0.556	0.580
TC (mmol/L)	0.431	0.832	4.129	0.077
TG (mmol/L)	−0.140	0.889	−4.014	< 0.001
HDL‐C (mmol/L)	−1.179	0.238	−1.189	0.234
LDL‐C (mmol/L)	0.936	0.679	2.910	0.008
MoCA‐BC	−1.744	0.081	−0.232	0.817

Abbreviations: LDL‐C, Low density lipoprotein cholesterol; MoCA‐BC, Montreal cognitive assessment basic edition score; MBP, Myelin basic protein; TC, Total cholesterol; TG, Triglycerides.

### 3.3 Association of Statin Therapy and Hyperlipidemia With Fazekas Score in CSVD Patients

After adjusting for hypertension, which showed significant inter‐group differences, the subjects were grouped based on whether they received statin treatment. The Fazekas score exhibited significant inter‐group differences only in the CSVD group (p = 0.018). Grouping based on the presence or absence of hyperlipidemia did not reveal any statistically significant differences. Further evaluation of the correlation between indicators such as TC, TG, HDL‐C, LDL‐C, and Fazekas score also did not indicate any statistically significant differences (Table 3).

**TABLE 3:** Association of Fazekas score with statin therapy, hyperlipidemia, and blood lipid levels in patients with cerebral small vessel disease.

							95% C.I.	
		B	S.E.	Exp(B)/Beta	Wald/t	Sig.	Lower	Upper
Statin therapy	CSVD	0.660	0.279	1.935	5.610	0.018	1.121	3.342
	NCSVD	−0.866	0.892	0.421	0.942	0.332	0.073	2.418
Hyperlipidemia	CSVD	0.121	0.224	1.128	0.292	0.589	0.728	1.749
	NCSVD	−0.768	0.554	0.464	1.925	0.165	0.157	1.373
TC	CSVD	−0.116	0.097	−0.137	−1.197	0.235	−0.308	0.077
	NCSVD	0.212	0.224	0.115	0.946	0.348	−0.235	0.658
TG	CSVD	−0.043	0.088	−0.059	−0.483	0.631	−0.218	0.133
	NCSVD	0.286	0.187	0.184	1.531	0.130	−0.087	0.659
HDL‐C	CSVD	−0.033	0.030	−0.120	−1.091	0.279	−0.094	0.027
	NCSV	−0.122	0.092	−0.160	−1.330	0.188	−0.306	0.061
LDL‐C	CSVD	−0.092	0.073	−0.147	−1.261	0.212	−0.237	0.053
	NCSVD	0.376	0.233	0.193	1.613	0.111	−0.089	0.841

Abbreviations: HDL‐C, High density lipoprotein cholesterol; LDL‐C, Low density lipoprotein cholesterol; TC, Total cholesterol; TG: Triglycerides.

Models were adjusted for hypertension.

Furthermore, in a subgroup analysis of CSVD patients, investigation into the association between statin therapy and various CSVD markers revealed that only WMH, which is related to cerebral white matter damage, demonstrated an association with statin use, while lacunes, ePVS, and CMBs did not show significant associations (Table 4).

**TABLE 4:** Association between statin therapy and various CSVD markers in the CSVD group.

	B	S.E.	Exp(B)	Wald	Sig.	95% C.I.	
						Lower	Upper
Fazekas score	0.661	0.328	1.938	4.056	0.044	1.018	3.688
Lacunes	−0.081	0.451	0.922	0.032	0.858	0.381	2.234
MARS score	0.159	0.347	1.172	0.209	0.648	0.593	2.314
ePVS(CSO)	−0.579	0.783	0.561	0.546	0.460	0.121	2.600
ePVS(BG)	0.723	0.808	2.061	0.801	0.371	0.423	10.039

Abbreviations: ePVS(BG), Enlarged perivascular spaces in basal ganglia; ePVS(CSO): Enlarged perivascular spaces in centrum semiovale; MARS: Microbleed Anatomical Rating Scale.

Models were adjusted for hypertension.

### 3.4 Association of Statin Therapy and Hyperlipidemia With Serum MBP Levels in CSVD Patients

Additionally, we observed that the subjects' serum MBP levels demonstrated similar patterns to WMH. Significant inter‐group differences related to statin use were only observed in the CSVD group (p = 0.006). Similarly, this difference was not evident in the grouping based on the presence or absence of hyperlipidemia, and no significant associations were found with TC, TG, or LDL levels (Table 5).

**TABLE 5:** Association of serum MBP levels with statin therapy, hyperlipidemia, and blood lipid levels in patients with cerebral small vessel disease.

				Exp(B)/Beta	Wald/t		95% C.I.	
		B	S.E.			Sig.	Lower	Upper
Statin therapy	CSVD	0.578	0.210	1.782	7.599	0.006	1.182	2.687
	NCSVD	0.234	0.253	1.264	0.854	0.355	0.769	2.077
Hyperlipidemia	CSVD	−0.075	0.161	0.928	0.218	0.640	0.677	1.271
	NCSVD	0.062	0.149	1.064	0.173	0.677	0.795	1.425
TC	CSVD	−0.030	0.073	−0.047	−0.411	0.682	−0.176	0.116
	NCSVD	0.004	0.058	0.008	0.068	0.946	−0.113	0.121
TG	CSVD	−0.064	0.066	−0.117	−0.968	0.336	−0.195	0.068
	NCSVD	−0.060	0.049	−0.150	−1.224	0.225	−0.157	0.038
HDL‐C	CSVD	0.032	0.023	0.155	1.411	0.163	−0.013	0.077
	NCSV	0.039	0.024	0.202	1.659	0.102	−0.008	0.087
LDL‐C	CSVD	−0.048	0.055	−0.104	−0.878	0.383	−0.158	0.061
	NCSVD	−0.009	0.062	−0.019	−0.153	0.879	−0.133	0.114

Abbreviations: HDL‐C, High density lipoprotein cholesterol; LDL‐C, Low density lipoprotein cholesterol; TC, Total cholesterol; TG, Triglycerides.

Models were adjusted for hypertension.

### 3.5 Association of Statin Therapy and Hyperlipidemia With Volume of the Fimbria in CSVD Patients

Furthermore, within the hippocampal formation, the volume of the fimbria, which is the sole structure predominantly composed of white matter, exhibited a correlation with statin use exclusively in the CSVD group; no such association was observed when patients were stratified by the presence of hyperlipidemia. With the exception of the right parasubiculum, none of the other non‐white matter regions exhibited significant volumes associated with statin use or the presence of hyperlipidemia. These findings were significant prior to, but did not survive, correction for multiple comparisons and should, therefore, be interpreted as exploratory (Table 6, Supplementary Table S1).

**TABLE 6:** Association of statin therapy and hyperlipidemia with volume of the fimbria in CSVD patients.

							95% C.I.	
		B	S.E.	Exp(B)	Wald	Sig.	Lower	Upper
		Left fimbria						
Statin therapy	CSVD	−0.042	0.016	0.959	6.903	0.009	0.929	0.989
	NCSVD	−0.023	0.030	0.977	0.620	0.431	0.922	1.035
Hyperlipidemia	CSVD	0.001	0.013	0.999	0.007	0.934	0.974	1.025
	NCSVD	−0.017	0.018	0.984	0.802	0.371	0.949	1.020
		Right fimbria						
Statin therapy	CSVD	−0.048	0.018	0.954	6.715	0.010	0.920	0.988
	NCSVD	0.016	0.034	1.017	0.230	0.632	0.950	1.088
Hyperlipidemia	CSVD	−0.011	0.016	0.990	0.447	0.504	0.959	1.021
	NCSVD	−0.033	0.021	0.968	2.328	0.127	0.928	1.009

Abbreviations: LDL‐C, Low density lipoprotein cholesterol; TC, Total cholesterol; TG, Triglycerides.

Models were adjusted for hypertension.

## 4 Discussion

This study found that age and hypertension remain the most significant risk factors for CSVD. As the study cohort excluded patients using statins for central nervous system indications, the intergroup differences observed in statin use likely originated from their prescriptions for managing conditions such as hypertension and atherosclerosis and thus may lack clinical significance. The key finding of this research is that, specifically within the CSVD group, statin therapy demonstrated significant associations with Fazekas scores, serum myelin basic protein levels, and bilateral hippocampal fimbria volume. This confirms a protective effect of statin treatment on white matter integrity exclusively in patients with CSVD. In contrast, no association was observed with blood lipid levels. Moreover, the protective effect of statin treatment was primarily manifested in measures related to cerebral white matter.

The findings of this study help explain previously reported inconsistencies regarding the role of dyslipidemia in CSVD. Such discrepancies may stem from methodological variations. Some studies investigated hyperlipidemia's impact on CSVD using various blood lipid indicators without considering statin therapy (Ohwaki et al. 2013; Szcześniak et al. 2021), while others combined statin use and hyperlipidemia as a single factor (Kohara et al. 2003). Studies that separately analyzed statin use and hyperlipidemia demonstrated that statin use was associated with less progression of WMH, independent of its lipid‐lowering effect (Xiong et al. 2014). This presents an intriguing paradox: although statins are primarily used to regulate peripheral blood lipids, their protective effect on white matter appears unrelated to peripheral lipid levels.

Furthermore, we observed that the protective effect of statins was evident only in the CSVD group. This suggests that their protection depends on the pathological changes of CSVD, especially BBB disruption. Under physiological conditions, cholesterol in the brain is synthesized almost entirely locally. Because the intact BBB isolates the brain, peripheral cholesterol has little influence on cerebral cholesterol homeostasis (Dietschy and Turley 2001). After BBB disruption, although cholesterol from the peripheral circulation can enter the brain, cholesterol synthesis within the brain increases by approximately 60%, which may be related to the efflux of brain‐derived 24‐hydroxycholesterol (Saeed et al. 2014). On the other hand, following BBB disruption, the resulting entry of statins allows for the re‐inhibition of brain cholesterol synthesis and the restoration of its homeostasis, ultimately generating a protective effect.

The association between MBP levels and statin use in CSVD provides further support and explanation for these findings. MBP is a crucial component of myelin, a specialized plasma membrane structure maintained by non‐covalent interactions between lipids and MBP (Min et al. 2009; Harauz et al. 2009). This interaction is influenced by membrane cholesterol content, with physiological 44% cholesterol content providing optimal binding (Träger et al. 2020). In CSVD, accompanying BBB disruption, enhanced brain cholesterol synthesis inevitably alters plasma membrane cholesterol proportions due to increased cholesterol content along the secretory pathway in eukaryotic cells (Hannich et al. 2011). This may destabilize myelin structure by reducing non‐covalent interactions between plasma membranes and MBP. Statin therapy can downregulate brain cholesterol synthesis, maintain appropriate plasma membrane cholesterol levels despite BBB disruption, preserve myelin structural integrity, and consequently reduce serum MBP levels.

Furthermore, we observed that statin therapy in CSVD patients also exhibited a protective effect on hippocampal fimbria volume. The hippocampal fimbria is a white matter ridge connecting the medial edge of the hippocampus, and its damage may lead to reduced effective information transmission between the hippocampus (HC) and the prefrontal cortex (PFC), resulting in impaired cognitive and memory functions (Henson et al. 2016). The protective effect of statins on the hippocampal fimbria may potentially prevent cognitive decline in CSVD patients and even slow the progression to cognitive impairment‐related diseases. This is particularly relevant in the context of the recently proposed CSVD‐AD continuum (van der Flier et al. 2018), where statin therapy shows potential in decelerating disease progression. These findings indicate that statin therapy might offer broader therapeutic potential in various conditions involving white matter damage secondary to BBB disruption, including cognitive impairment and dementia.

This protective mechanism of statin therapy against white matter injury may extend to other neurological disorders characterized by BBB dysfunction. Supporting this notion, a study demonstrated that rosuvastatin reduces white matter structural damage in stroke‐prone renovascular hypertensive rats, a model featuring impaired BBB tight junctions (Zheng et al. 2020). These findings suggest that statin therapy holds broader therapeutic potential for various conditions where BBB disruption contributes to secondary white matter injury.

Further basic experiments are needed to substantiate our findings, relying on established CSVD animal models with typical pathological features, methods for myelin membrane cholesterol content analysis (Larocca and Norton 2007), and specialized equipment for measuring intermembrane forces. Future research should focus on determining optimal timing and duration of statin therapy, comparing effectiveness among different statins, and developing specific markers for monitoring central nervous system cholesterol synthesis during treatment.

## Author Contributions

Rui Wang and Juluo Chen conceived and designed the study. Rui Wang performed data curation, formal analysis, visualization, and wrote the original draft. Juluo Chen, Rui Wang, Peiqi Ma, and Lu Liu conducted the investigation. Juluo Chen, Rui Wang, and Peiqi Ma developed the methodology. Project administration was handled by Juluo Chen, Rui Wang, Youmeng Wang, and Qiqiang Tang. Resources were provided by Youmeng Wang and Qiqiang Tang. Supervision was carried out by Youmeng Wang and Qiqiang Tang. Rui Wang was responsible for reviewing and editing the manuscript. All authors reviewed and approved the final manuscript.

## Funding

This work was supported by the National Natural Science Foundation of China (81573807).

## Ethics Statement

The study was approved by the Ethics Committee of Fuyang People's Hospital (approval number: Medical [2022] No. 26).

## Conflicts of Interest

The authors declare no conflicts of interest.

## Supporting information




**Supplementary Table**: brb371359‐sup‐0001‐TableS1.docx

## Data Availability

The datasets generated and analyzed during the current study contain sensitive personal information that could compromise research participant privacy/consent. Therefore, the full dataset cannot be made publicly available. However, de‐identified data that support the findings of this study are available from the corresponding author upon reasonable request and with approval from the Ethics Committee of Fuyang People's Hospital.
