# Supplementary material for: Statin Therapy Protects White Matter in Cerebral Small Vessel Disease Independent of Lipid‐Lowering: Biomarker and Neuroimaging Evidence
Source: Brain Behav. 2026 May 11;16(5):e71359. doi: 10.1002/brb3.71359 (PMC13159140; doi:10.1002/brb3.71359)
Supplement: Supplementary file 1 — Supplementary Table: brb371359‐sup‐0001‐TableS1.docx [file BRB3-16-e71359-s001.docx]

Table S1 Association of Statin Use and Hyperlipidemia with Hippocampal Subfields

|  |  | B | S.E. | Exp(B) | Wald | Sig. | 95% C.I. | |
| --- | --- | --- | --- | --- | --- | --- | --- | --- |
|  |  |  |  |  |  |  | Lower | Upper |
| Left Hippocampal tail | | | | | | | | |
| Statin therapy | CSVD | 0.000 | 0.004 | 1.000 | 0.000 | 0.989 | 0.993 | 1.007 |
|  | NCSVD | 0.007 | 0.007 | 1.007 | 1.132 | 0.287 | 0.994 | 1.021 |
| Hyperlipidemia | CSVD | -0.001 | 0.004 | 0.999 | 0.127 | 0.722 | 0.992 | 1.006 |
|  | NCSVD | -0.013 | 0.004 | 0.988 | 7.784 | 0.005 | 0.979 | 0.996 |
| Right Hippocampal tail | | | | | | | | |
| Statin therapy | CSVD | 0.003 | 0.004 | 1.003 | 0.779 | 0.377 | 0.996 | 1.011 |
|  | NCSVD | 0.005 | 0.007 | 1.005 | 0.441 | 0.506 | 0.990 | 1.020 |
| Hyperlipidemia | CSVD | -0.002 | 0.004 | 0.998 | 0.401 | 0.527 | 0.991 | 1.005 |
|  | NCSVD | -0.008 | 0.004 | 0.992 | 3.554 | 0.059 | 0.984 | 1.000 |
| Left subiculum | | | | | | | | |
| Statin therapy | CSVD | 0.003 | 0.005 | 1.003 | 0.215 | 0.643 | 0.992 | 1.013 |
|  | NCSVD | 0.007 | 0.011 | 1.007 | 0.405 | 0.524 | 0.986 | 1.028 |
| Hyperlipidemia | CSVD | 0.001 | 0.005 | 1.001 | 0.023 | 0.880 | 0.991 | 1.011 |
|  | NCSVD | -0.003 | 0.006 | 0.997 | 0.177 | 0.674 | 0.985 | 1.010 |
| Right subiculum | | | | | | | | |
| Statin therapy | CSVD | 0.004 | 0.006 | 1.004 | 0.404 | 0.525 | 0.992 | 1.015 |
|  | NCSVD | -0.007 | 0.011 | 0.993 | 0.381 | 0.537 | 0.972 | 1.015 |
| Hyperlipidemia | CSVD | 0.007 | 0.006 | 1.007 | 1.351 | 0.245 | 0.996 | 1.018 |
|  | NCSVD | -0.007 | 0.007 | 0.993 | 1.002 | 0.317 | 0.979 | 1.007 |
| Left CA1 | | | | | | | | |
| Statin therapy | CSVD | 0.003 | 0.004 | 1.003 | 0.711 | 0.399 | 0.995 | 1.012 |
|  | NCSVD | -0.005 | 0.008 | 0.995 | 0.418 | 0.518 | 0.980 | 1.010 |
| Hyperlipidemia | CSVD | -0.002 | 0.004 | 0.998 | 0.209 | 0.648 | 0.991 | 1.006 |
|  | NCSVD | 0.001 | 0.004 | 1.001 | 0.032 | 0.858 | 0.992 | 1.009 |
| Right CA1 | | | | | | | | |
| Statin therapy | CSVD | 0.007 | 0.004 | 1.007 | 2.724 | 0.099 | 0.999 | 1.014 |
|  | NCSVD | -0.002 | 0.007 | 0.998 | 0.061 | 0.805 | 0.985 | 1.012 |
| Hyperlipidemia | CSVD | 0.000 | 0.004 | 1.000 | 0.000 | 0.994 | 0.993 | 1.007 |
|  | NCSVD | -0.002 | 0.004 | 0.998 | 0.227 | 0.634 | 0.990 | 1.006 |
| Left hippocampalfissure | | | | | | | | |
| Statin therapy | CSVD | 0.008 | 0.009 | 1.008 | 0.765 | 0.382 | 0.990 | 1.026 |
|  | NCSVD | -0.010 | 0.023 | 0.990 | 0.194 | 0.659 | 0.945 | 1.036 |
| Hyperlipidemia | CSVD | -0.007 | 0.009 | 0.993 | 0.567 | 0.452 | 0.976 | 1.011 |
|  | NCSVD | -0.017 | 0.015 | 0.983 | 1.330 | 0.249 | 0.954 | 1.012 |
| Right hippocampalfissure | | | | | | | | |
| Statin therapy | CSVD | 0.004 | 0.009 | 1.004 | 0.214 | 0.643 | 0.987 | 1.021 |
|  | NCSVD | -0.015 | 0.018 | 0.985 | 0.665 | 0.415 | 0.950 | 1.021 |
| Hyperlipidemia | CSVD | 0.012 | 0.009 | 1.012 | 1.703 | 0.192 | 0.994 | 1.029 |
|  | NCSVD | 0.013 | 0.012 | 1.013 | 1.186 | 0.276 | 0.989 | 1.038 |
| Left presubiculum | | | | | | | | |
| Statin therapy | CSVD | -0.006 | 0.007 | 0.994 | 0.805 | 0.369 | 0.981 | 1.007 |
|  | NCSVD | -0.007 | 0.011 | 0.993 | 0.365 | 0.546 | 0.971 | 1.016 |
| Hyperlipidemia | CSVD | 0.006 | 0.007 | 1.006 | 0.825 | 0.364 | 0.993 | 1.019 |
|  | NCSVD | -0.009 | 0.007 | 0.991 | 1.482 | 0.223 | 0.977 | 1.006 |
| Right presubiculum | | | | | | | | |
| Statin therapy | CSVD | -0.005 | 0.007 | 0.995 | 0.515 | 0.473 | 0.981 | 1.009 |
|  | NCSVD | 0.016 | 0.015 | 1.016 | 1.118 | 0.290 | 0.986 | 1.047 |
| Hyperlipidemia | CSVD | 0.012 | 0.008 | 1.012 | 2.374 | 0.123 | 0.997 | 1.028 |
|  | NCSVD | -0.010 | 0.010 | 0.990 | 1.005 | 0.316 | 0.971 | 1.009 |
| Left parasubiculum | | | | | | | | |
| Statin therapy | CSVD | -0.019 | 0.018 | 0.981 | 1.076 | 0.300 | 0.947 | 1.017 |
|  | NCSVD | -0.043 | 0.034 | 0.958 | 1.655 | 0.198 | 0.897 | 1.023 |
| Hyperlipidemia | CSVD | 0.051 | 0.023 | 1.053 | 4.901 | 0.027 | 1.006 | 1.101 |
|  | NCSVD | -0.003 | 0.021 | 0.997 | 0.018 | 0.894 | 0.957 | 1.039 |
| Right parasubiculum | | | | | | | | |
| Statin therapy | CSVD | -0.052 | 0.024 | 0.949 | 4.830 | 0.028 | 0.906 | 0.994 |
|  | NCSVD | -0.013 | 0.053 | 0.987 | 0.061 | 0.804 | 0.890 | 1.095 |
| Hyperlipidemia | CSVD | 0.035 | 0.025 | 1.036 | 2.038 | 0.153 | 0.987 | 1.088 |
|  | NCSVD | -0.021 | 0.028 | 0.980 | 0.542 | 0.461 | 0.927 | 1.035 |
| Left molecular laye | | | | | | | | |
| Statin therapy | CSVD | 0.004 | 0.005 | 1.004 | 0.562 | 0.454 | 0.994 | 1.014 |
|  | NCSVD | 0.000 | 0.009 | 1.000 | 0.000 | 0.990 | 0.983 | 1.018 |
| Hyperlipidemia | CSVD | -0.001 | 0.005 | 0.999 | 0.075 | 0.784 | 0.990 | 1.008 |
|  | NCSVD | -0.003 | 0.006 | 0.997 | 0.341 | 0.559 | 0.986 | 1.008 |
| Right molecular layer | | | | | | | | |
| Statin therapy | CSVD | 0.006 | 0.005 | 1.006 | 1.468 | 0.226 | 0.996 | 1.016 |
|  | NCSVD | -0.004 | 0.010 | 0.996 | 0.156 | 0.693 | 0.977 | 1.015 |
| Hyperlipidemia | CSVD | 0.001 | 0.004 | 1.001 | 0.050 | 0.823 | 0.992 | 1.010 |
|  | NCSVD | -0.005 | 0.006 | 0.995 | 0.605 | 0.437 | 0.984 | 1.007 |
| Left GC-ML-DG | | | | | | | | |
| Statin therapy | CSVD | 0.001 | 0.009 | 1.001 | 0.028 | 0.868 | 0.985 | 1.018 |
|  | NCSVD | -0.006 | 0.018 | 0.994 | 0.115 | 0.735 | 0.959 | 1.030 |
| Hyperlipidemia | CSVD | -0.004 | 0.008 | 0.996 | 0.308 | 0.579 | 0.981 | 1.011 |
|  | NCSVD | -0.003 | 0.011 | 0.997 | 0.077 | 0.782 | 0.977 | 1.018 |
| Right GC-ML-DG | | | | | | | | |
| Statin therapy | CSVD | 0.005 | 0.008 | 1.005 | 0.325 | 0.568 | 0.988 | 1.022 |
|  | NCSVD | -0.012 | 0.019 | 0.988 | 0.429 | 0.512 | 0.952 | 1.025 |
| Hyperlipidemia | CSVD | -0.002 | 0.008 | 0.998 | 0.069 | 0.793 | 0.983 | 1.013 |
|  | NCSVD | -0.006 | 0.011 | 0.994 | 0.318 | 0.573 | 0.973 | 1.015 |
| Left CA3 | | | | | | | | |
| Statin therapy | CSVD | 0.016 | 0.010 | 1.016 | 2.772 | 0.096 | 0.997 | 1.036 |
|  | NCSVD | -0.003 | 0.020 | 0.997 | 0.018 | 0.893 | 0.959 | 1.037 |
| Hyperlipidemia | CSVD | -0.010 | 0.009 | 0.990 | 1.329 | 0.249 | 0.973 | 1.007 |
|  | NCSVD | 0.001 | 0.012 | 1.001 | 0.002 | 0.961 | 0.978 | 1.024 |
| Right CA3 | | | | | | | | |
| Statin therapy | CSVD | 0.015 | 0.010 | 1.015 | 2.271 | 0.132 | 0.996 | 1.035 |
|  | NCSVD | -0.019 | 0.021 | 0.981 | 0.819 | 0.365 | 0.942 | 1.022 |
| Hyperlipidemia | CSVD | -0.009 | 0.009 | 0.991 | 0.932 | 0.334 | 0.973 | 1.009 |
|  | NCSVD | -0.003 | 0.011 | 0.997 | 0.075 | 0.784 | 0.976 | 1.019 |
| Left CA4 | | | | | | | | |
| Statin therapy | CSVD | 0.007 | 0.011 | 1.007 | 0.385 | 0.535 | 0.986 | 1.027 |
|  | NCSVD | -0.010 | 0.022 | 0.990 | 0.219 | 0.640 | 0.948 | 1.033 |
| Hyperlipidemia | CSVD | -0.005 | 0.009 | 0.996 | 0.239 | 0.625 | 0.978 | 1.014 |
|  | NCSVD | -0.006 | 0.013 | 0.994 | 0.247 | 0.619 | 0.970 | 1.018 |
| Right CA4 | | | | | | | | |
| Statin therapy | CSVD | 0.007 | 0.010 | 1.007 | 0.440 | 0.507 | 0.987 | 1.027 |
|  | NCSVD | -0.014 | 0.022 | 0.986 | 0.383 | 0.536 | 0.945 | 1.030 |
| Hyperlipidemia | CSVD | 0.000 | 0.009 | 1.000 | 0.000 | 0.990 | 0.982 | 1.019 |
|  | NCSVD | -0.006 | 0.012 | 0.994 | 0.251 | 0.617 | 0.970 | 1.018 |
| Left HATA | | | | | | | | |
| Statin therapy | CSVD | -0.029 | 0.026 | 0.971 | 1.259 | 0.262 | 0.922 | 1.022 |
|  | NCSVD | 0.014 | 0.055 | 1.014 | 0.067 | 0.796 | 0.911 | 1.130 |
| Hyperlipidemia | CSVD | -0.006 | 0.025 | 0.994 | 0.063 | 0.802 | 0.946 | 1.044 |
|  | NCSVD | 0.013 | 0.034 | 1.013 | 0.154 | 0.694 | 0.948 | 1.083 |
| Right HATA | | | | | | | | |
| Statin therapy | CSVD | 0.006 | 0.031 | 1.006 | 0.034 | 0.854 | 0.946 | 1.069 |
|  | NCSVD | 0.063 | 0.062 | 1.065 | 1.009 | 0.315 | 0.942 | 1.203 |
| Hyperlipidemia | CSVD | -0.071 | 0.032 | 0.931 | 4.923 | 0.027 | 0.875 | 0.992 |
|  | NCSVD | -0.020 | 0.035 | 0.980 | 0.331 | 0.565 | 0.915 | 1.050 |
| Left fimbria | | | | | | | | |
| Statin therapy | CSVD | -0.042 | 0.016 | 0.959 | 6.903 | 0.009 | 0.929 | 0.989 |
|  | NCSVD | -0.023 | 0.030 | 0.977 | 0.620 | 0.431 | 0.922 | 1.035 |
| Hyperlipidemia | CSVD | -0.001 | 0.013 | 0.999 | 0.007 | 0.934 | 0.974 | 1.025 |
|  | NCSVD | -0.017 | 0.018 | 0.984 | 0.802 | 0.371 | 0.949 | 1.020 |
| Right fimbria | | | | | | | | |
| Statin therapy | CSVD | -0.048 | 0.018 | 0.954 | 6.715 | 0.010 | 0.920 | 0.988 |
|  | NCSVD | 0.016 | 0.034 | 1.017 | 0.230 | 0.632 | 0.950 | 1.088 |
| Hyperlipidemia | CSVD | -0.011 | 0.016 | 0.990 | 0.447 | 0.504 | 0.959 | 1.021 |
|  | NCSVD | -0.033 | 0.021 | 0.968 | 2.328 | 0.127 | 0.928 | 1.009 |
